# Comprehensive Calibration of Strap-Down Tri-Axis Accelerometer Unit

**DOI:** 10.3390/mi8030068

**Published:** 2017-02-27

**Authors:** Xi Zhang, Jie Li, Li Qin, Chong Shen

**Affiliations:** 1 National Key Laboratory for Electronic Measurement Technology, North University of China, Taiyuan 030051, China; zhangxi@nuc.edu.cn (X.Z.); qinli@nuc.edu.cn (L.Q.); 2 School of Computer Science and Control Engineering, North University of China, Taiyuan 030051, China; 3 School of Instrument and Electronics, North University of China, Taiyuan 030051, China; shenchong@nuc.edu.cn; 4 Key Laboratory of Instrumentation Science & Dynamic Measurement, Ministry of Education, North University of China, Taiyuan 030051, China

**Keywords:** strap-down tri-axis accelerometer, static error, dynamic error, calibration

## Abstract

This paper proposes a comprehensive calibration method to improve the precision of a strap-down tri-axis accelerometer unit, in which parameters are divided into static and dynamic ones. The contribution of the manuscript is that it solves the problem of inappropriate installation and the size effect error for tri-axis accelerometer unit at high speed by using static and dynamic calibration method, respectively. The experiment results show the measuring accuracy of accelerometers is increased by more than one order of magnitude, and the navigation precision is increased by more than two orders of magnitude.

## 1. Introduction

For strap-down Inertial Navigation System (SINS), an inertial measurement unit (IMU) is directly installed in the vehicle. Thus, the navigation precision of SINS depends on the measuring accuracy of IMU [1,2,3,4,5,6]. In practical application, installation of accelerometer and gyro is not orthogonal completely, and the installation error will arise. Take IMU as a rigid body. If we calibrate the orthogonal error correctly, the angular velocity can be measured regardless of position and orientation of the gyro. However, the different positions in IMU have different accelerations when it rotates. This phenomenon is the so-called size effect error or the lever arm effect error for accelerometer, which is caused by the installation position error of IMU.

Now, the existing calibration methods generally use a static model to compensate errors of the strap-down tri-axis accelerometer unit [7,8,9,10,11,12,13,14,15,16,17,18,19,20,21]. A multi-position calibration method was designed for microelectromechanical systems (MEMS) of high to medium quality, and the excellent performance of the proposed method was proved by experimental results [8,9]. Methods were presented to calibrate and compensate for non-zero biases, non-unit scale factors, axis misalignments and cross-axis sensitivities of both the tri-axial accelerometer and gyroscopic setups in a MEMS based IMU, which depended on the Earth’s gravity as a stable physical calibration standard. In addition, experimental results showed that, with calibration, the observed average static angular error is less than a quarter of a degree and the dynamic angular error is reduced effectively [9]. An optical calibration method to address the alignment problem was proposed, and the experiments were performed with a MEMS-based azimuth-level detector to show the effectiveness of the proposed method [6]. Under the low dynamic environment, the size effect error is relatively small and can be ignored, and the above methods are effective. However, the effect of the size error becomes serious at high angular velocity, and the navigation error caused by it should not be neglected. Therefore, we need to explore a more accurate calibration method.

In this paper, we propose a comprehensive calibration method. In this method, we assume that the position in which the specific force load on the accelerometer unit is a point, and hence a new model of dynamic error calibration is set up based on this method. The proposed method includes 15 parameters. In the parameters, there are three zero bias voltages, nine installation angles and three related factors, respectively. The errors of the accelerometer are completely separated through calibration of 15 parameters, which can fully reflect the characteristics of the sensor itself and improve measurement accuracy of the sensor. The proposed method makes the coordinate system of the accelerometer correspondent to the standard orthogonal coordinate system of carrier. After calibration, it can make three installation directions of the accelerometer completely equivalent to the standard coordinate system and ensure the accelerometer unit better perpendicularity.

A list of nomenclature is given as follows:

(1) SINS: strap-down inertial navigation system;
(2) IMU: inertial measurement unit;
(3) MIMU: MEMS inertial measurement unit.

## 2. Calibration Model

Accuracy of the calibration is the premise of compensating of tri-accelerometer unit. Here, we define the coordinate system as the following:

(1) Body coordinate frame (b-frame): its origin O_b_ is the center of IMU, three orthogonal axials are represented as *x*_b_, *y*_b_ and *z*_b_, respectively, and (*r_x_* *r_y_* *r_z_*) is the distance between position of the accelerometer and origin O, respectively.
(2) Sensitive axis coordinate frame (a-frame): *x*_a_, *y*_a_ and *z*_a_ represent a three-coordinate axis in an a-frame.
(3) Navigation coordinate system (n-frame): its origin O is at the center of the gravity vector.

### 2.1. Calibration Model of Static Error

**Figure 1:**
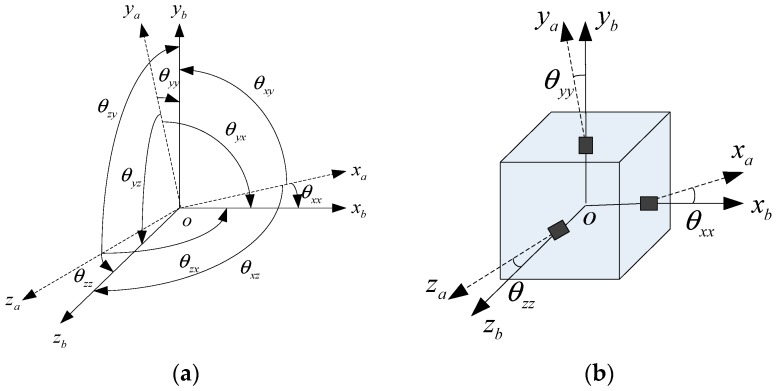
Model of static calibrate for accelerometer unit: (**a**) angles between a-frame and b-frame; (**b**) installation of tri-accelerometer unit.

In this paper, the installation position error is defined as the relative location between the actual sensitive spot of each accelerometer and the coordinate origin of the IMU structure. In order to reduce the output error, the installation errors should be compensated, and the accelerometer output should be transformed from a-frame to b-frame. The coordinate systems mentioned above are shown in Figure 1.

Figure 1 represents the hexahedral reference orthogonal coordinate system O_b_*x*_b_*y*_b_*z*_b_ of MIMU, and the non-orthogonal coordinate system, which is the actual measuring direction for accelerometers. For simplicity, O_b_ and O_a_ are set at the same point O.

In Figure 1, θ*_xx_*, θ*_xy_*, θ*_xz_*, θ*_yx_*, θ*_yy_*, θ*_yz_*, θ*_zx_*, θ*_zy_* and θ*_zz_* are installation angles for tri-accelerometers unit, and θ*_ij_* is the angle between a*_i_* and b*_j_*. 

$k_{x}^{a}$, $k_{y}^{a}$ and $k_{z}^{a}$ are scale factors of three accelerometers. $u_{x0}^{a}$, $u_{y0}^{a}$, and $u_{z0}^{a}$ are zero bias voltage. *x*_b_, *y*_b_, and *z*_b_ are three axial directions of reference frame, $f_{x}^{b}$, $f_{y}^{b}$, and $f_{z}^{b}$ are a specific force in reference frame b, *x*_a_, *y*_a_, and *z*_a_ are actual axial directions, and $f_{x}^{a}$, $f_{y}^{a}$, and $f_{z}^{a}$ are a specific force in reference frame a. 

The output $u_{i}^{a}$ of $A_{i}$ in a-frame can be given in Equation (1)

(1) $$u_{i}^{a}=k_{i}^{a}f_{i}^{a}+u_{i0}^{a}+\delta_{i}=k_{i}^{a}\left(f_{i}^{a}+B_{i}^{a}\right)+\delta_{i},\left(i=x,y,z\right)$$

Here, $u_{i0}^{a}=k_{i}^{a}\cdot B_{i}^{a}$, $B_{i}^{a}=\frac{u_{i0}^{a}}{k_{i}^{a}}$, $u_{i}^{a}$, $k_{i}^{a}$, $B_{i}^{a}$, $f_{i}^{a}$ are output voltage, scale factors, zero bias, and actual specific force input, respectively. The noise error $\delta_{i}$ can be ignored. Equation (1) can be simplified as:

(2) $$u_{i}^{a}=k_{i}^{a}f_{i}^{a}+u_{i0}^{a}=k_{i}^{a}\left(f_{i}^{a}+B_{i}^{a}\right),\left(i=x,y,z\right)$$

If we set $C_{a}^{b}$ as a transition matrix from a to b reference frame, specific forces can be changed from an a to b reference frame followed by $f^{b}=C_{a}^{b}f^{a}$, namely

(3) $$f^{b}=\left[\begin{array}{c} {f_{x}}^{b}\\ {f_{y}}^{b}\\ {f_{z}}^{b} \end{array}\right]=\left[\begin{array}{ccc} C_{a}^{b}\left(1,1\right) & C_{a}^{b}\left(1,2\right) & C_{a}^{b}\left(1,3\right)\\ C_{a}^{b}\left(2,1\right) & C_{a}^{b}\left(2,2\right) & C_{a}^{b}\left(2,3\right)\\ C_{a}^{b}\left(3,1\right) & C_{a}^{b}\left(3,2\right) & C_{a}^{b}\left(3,3\right) \end{array}\right]\left[\begin{array}{c} {f_{x}}^{a}\\ {f_{y}}^{a}\\ {f_{z}}^{a} \end{array}\right]=C_{a}^{b}f^{a}$$

Because the reference frame b is orthogonal, and $C_{a}^{b}$ is an orthogonal matrix, $f^{a}$ can be given by

(4) $$\begin{array}{l} f^{a}=C_{b}^{a}f^{b}={\left(C_{a}^{b}\right)}^{-1}f^{b}={\left(C_{a}^{b}\right)}^{T}f^{b}\\ =\left[\begin{array}{ccc} C_{a}^{b}\left(1,1\right) & C_{a}^{b}\left(2,1\right) & C_{a}^{b}\left(3,1\right)\\ C_{a}^{b}\left(1,2\right) & C_{a}^{b}\left(2,2\right) & C_{a}^{b}\left(3,2\right)\\ C_{a}^{b}\left(1,3\right) & C_{a}^{b}\left(2,3\right) & C_{a}^{b}\left(3,3\right) \end{array}\right]\left[\begin{array}{c} {f_{x}}^{b}\\ {f_{y}}^{b}\\ {f_{z}}^{b} \end{array}\right] \end{array}$$

Substituting Equation (4) into Equation (2), $u_{i}^{a}$ is simplified as Equation (5)

(5) $$u_{i}^{a}=\left[\begin{array}{c} u_{x}^{a}\\ u_{y}^{a}\\ u_{z}^{a} \end{array}\right]=\left[\begin{array}{ccc} k_{x} & 0 & 0\\ 0 & k_{y} & 0\\ 0 & 0 & k_{z} \end{array}\right]\left[\begin{array}{c} {f_{x}}^{a}\\ {f_{y}}^{a}\\ {f_{z}}^{a} \end{array}\right]+\left[\begin{array}{c} u_{x0}^{a}\\ u_{y0}^{a}\\ u_{z0}^{a} \end{array}\right]=K{\left(C_{a}^{b}\right)}^{T}f^{b}+u_{i0}^{a}$$

From Equation (5), $f^{b}$ are obtained in Equation (6)

(6) $$\begin{array}{l} f^{b}={\left[K\cdot {\left(C_{a}^{b}\right)}^{T}\right]}^{-1}\left(u_{i}^{a}-u_{i0}^{a}\right)\\ ={\left[{\left(C_{a}^{b}\right)}^{T}\right]}^{-1}\left[K^{-1}\left(u_{i}^{a}-u_{i0}^{a}\right)\right]=K_{A}\cdot U_{A}-\nabla^{b} \end{array}$$

Because $U_{A}={\left(\begin{array}{ccc} u_{x}^{a} & u_{y}^{a} & u_{z}^{a} \end{array}\right)}^{T}$ are the results from three accelerometers, $K_{A}$ is scale factor, and ${K_{A}}^{-1}$ is given by Equation (7)

(7) $$\begin{array}{l} {K_{A}}^{-1}=K\cdot {\left(C_{a}^{b}\right)}^{T}\\ =\left[\begin{array}{ccc} k_{x}C_{a}^{b}\left(1,1\right) & k_{x}C_{a}^{b}\left(2,1\right) & k_{x}C_{a}^{b}\left(3,1\right)\\ k_{y}C_{a}^{b}\left(1,2\right) & k_{y}C_{a}^{b}\left(2,2\right) & k_{y}C_{a}^{b}\left(3,2\right)\\ k_{z}C_{a}^{b}\left(1,3\right) & k_{z}C_{a}^{b}\left(2,3\right) & k_{z}C_{a}^{b}\left(3,3\right) \end{array}\right] \end{array}$$

$B={\left(\begin{array}{ccc} B_{x}^{a} & B_{y}^{a} & B_{z}^{a} \end{array}\right)}^{T}$ is a vector of zero bias specific force, and then the equivalent zero bias vector $\nabla^{b}$ is given by Equation (8):

(8) $$\begin{array}{l} \nabla^{b}={\left[{\left(C_{a}^{b}\right)}^{T}\right]}^{-1}\left[{\left(K\right)}^{-1}u_{i0}^{a}\right]\\ ={\left[{\left(C_{a}^{b}\right)}^{T}\right]}^{-1}\left[{\left(K\right)}^{-1}k_{i}^{a}B_{i}^{a}\right]={\left[{\left(C_{a}^{b}\right)}^{T}\right]}^{-1}B \end{array}$$

$C_{a}^{b}\left(*,j\right)$ is the unit column vector of the conversion matrix, and its element value is the direction cosine of $A_{j}$ and the unit vectors $i_{x}^{b}$, $i_{y}^{b}$, $i_{z}^{b}$, that is, Equation (9):

(9) $$C_{a}^{b}\left(*,j\right)=\left[\begin{array}{ccc} {\cos\theta }_{xx} & {\cos\theta }_{yx} & {\cos\theta }_{zx}\\ {\cos\theta }_{xy} & {\cos\theta }_{yy} & {\cos\theta }_{zy}\\ {\cos\theta }_{xz} & {\cos\theta }_{yz} & {\cos\theta }_{zz} \end{array}\right]$$

The results of **K**, $C_{a}^{b}$, $u_{i0}$, $B_{i0}$ and $f^{b}$, which are known from Equations (6), (8) and (9) can be obtained by using multi-position static turntable calibration tests. 

Measurement reference point of specific force $f^{b}$ should be at a b coordinate origin. However, the static calibration can only determine the direction of accelerometer and can not determine the actual sensitive point. Therefore, the dynamic calibration is necessary during measurement.

### 2.2. Calibration Model of Dynamic Error

**Figure 2:**
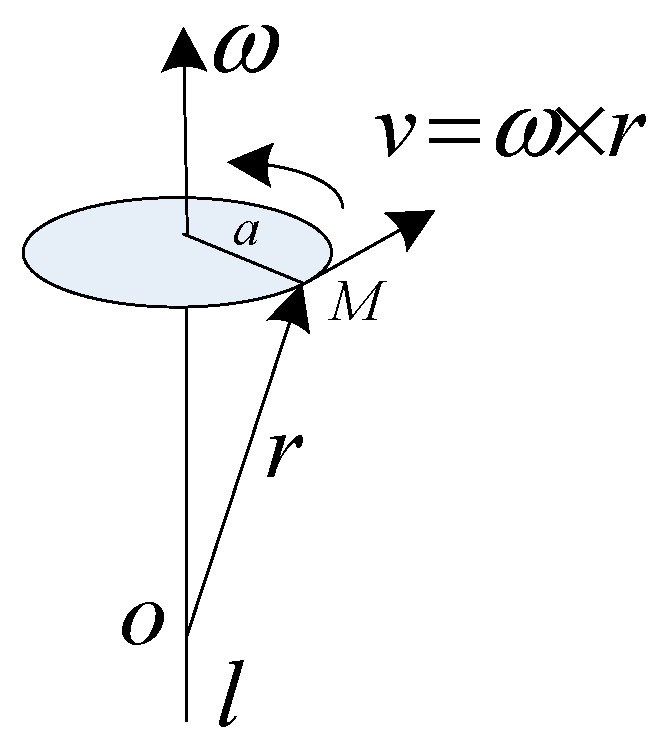
Diagram of dynamic error caused by the size effect.

Linear velocity of any point in rigid body is v=ω×r, as shown in Figure 2, a is the acceleration of some point, and then:

(10) $$\begin{array}{l} a=\frac{dv}{dt}=\frac{d\left(\omega \times r\right)}{dt}\\ =\frac{d\omega }{dt}\times r+\omega \times \frac{dr}{dt}=\overset{•}{\omega }\times r+\omega \times v\\ =\overset{•}{\omega }\times r+\omega \times \left(\omega \times r\right)=a_{t}+a_{n} \end{array}$$

$a_{t}$ is tangential acceleration, and $a_{n}$ is normal acceleration. MIMU is regarded as a rigid body. The acceleration of each point in the rigid body is different when the angular motion happens. The measurement point of the specific force is different from the existence of installation position error. It is necessary to compensate the dynamic error and make the measurement point equivalent to a point.

**Figure 3:**
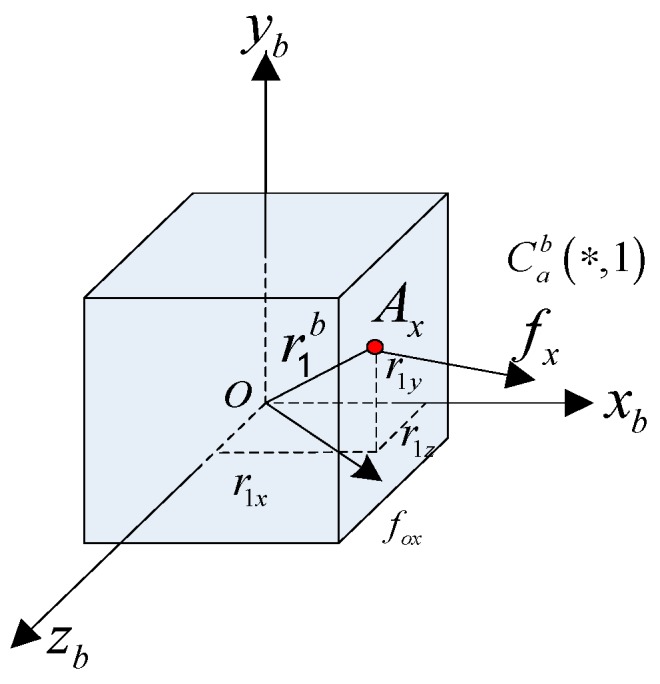
Model of dynamic calibrate for accelerometer $A_{x}$.

In Figure 3, accelerometer $A_{x}$ is taken as an example. $A_{x}$ is the sensitive point of an accelerometer, the frame $Ox_{b}y_{b}z_{b}$ is the calibration coordinate system (frame b), and point O is the reference point of the sensitive value. Installation position or sensitive direction of $A_{x}$ is $C_{a}^{b}\left(*,1\right)$. $r_{1}^{b}$ is the position vector from $A_{x}$ point to the origin of frame b.

Specific force at point $A_{x}$ is $f_{x}$, and specific force at point O is $f_{ox}$. When there is an angular motion, we can get the relationship between two specific forces in Equation (11)

(11) $$\begin{array}{l} f_{x}^{b}=f_{ox}^{b}+\overset{•}{\omega^{b}}\times r_{1}^{b}+\omega^{b}\times \left(\omega^{b}\times r_{1}^{b}\right)\\ =f_{ox}^{b}+\left[\overset{•}{\omega^{b}}\times +{\left(\omega^{b}\times \right)}^{2}\right]r_{1}^{b}=f_{ox}^{b}+Wr_{1}^{b} \end{array}$$

$W=\left(\overset{•}{\omega^{b}}\times \right)+{\left(\omega^{b}\times \right)}^{2}$, ω is angular velocity, $\overset{•}{\omega^{b}}\times r_{1}^{b}$, and $\omega^{b}\times \left(\omega^{b}\times r_{1}^{b}\right)$ are tangential acceleration and normal acceleration. (•×) is an anti-symmetric matrix that is formed by a vector, and ${\left(\omega^{b}\times \right)}^{2}$ is a symmetric matrix. The projections of installation position $r_{x}^{b}$ in frame b are $r_{1x}^{b}$, $r_{1y}^{b}$ and $r_{1z}^{b}$, Equation (11) is projected to the *x*-axis in frame a, that is, multiply $C_{a}^{b}{\left(*,1\right)}^{T}$ to both sides of Equation (12), and the specific force output of *x*-axis can be obtained:

(12) $$f_{X}=C_{a}^{b}{\left(*,1\right)}^{T}f_{x}^{b}=C_{a}^{b}{\left(*,1\right)}^{T}f_{ox}^{b}+C_{a}^{b}{\left(*,1\right)}^{T}Wr_{1}^{b}=f_{oX}+f_{XL}$$

$f_{X}$ is the specific force along the sensitive axis of sensor, $f_{oX}$ is the projection that is sensitive to specific force in frame b, and $f_{XL}$ is the specific force error caused by lever arm effect. Then, Equation (13) can be obtained:

(13) $$u_{X}=k_{X}\left(B_{X}+f_{X}\right)=k_{X}\left(B_{X}+f_{oX}^{a}+f_{XL}\right)$$

Similarly, the deduction of *y*-axis and *z*-axis can be obtained:

(14) $$\left[\begin{array}{c} u_{X}\\ u_{Y}\\ u_{Z} \end{array}\right]=\left[\begin{array}{ccc} k_{X} & 0 & 0\\ 0 & k_{Y} & 0\\ 0 & 0 & k_{Z} \end{array}\right]\cdot \left(\left[\begin{array}{c} B_{X}\\ B_{Y}\\ B_{Z} \end{array}\right]+\left[\begin{array}{c} f_{oX}^{a}\\ f_{oY}^{a}\\ f_{oY}^{a} \end{array}\right]+\left[\begin{array}{c} f_{XL}\\ f_{YL}\\ f_{ZL} \end{array}\right]\right)$$

Equation (14) can be simplified as

(15) $$U_{A}=K\left(B+f_{o}^{a}+f_{L}\right)$$

According to static calibration model $f^{a}={\left(C_{a}^{b}\right)}^{T}f^{b}$, we can obtain $U_{A}=K\left[B+{\left(C_{a}^{b}\right)}^{T}f^{b}+f_{L}\right]$.

Then,

(16) $$f^{b}={\left[{\left(C_{a}^{b}\right)}^{T}\right]}^{-1}\left[U_{A}{\left(K\right)}^{-1}-B-f_{L}\right]=K_{A}\cdot U_{A}-\nabla^{b}-f_{L}^{b}$$

$f_{L}^{b}={\left[{\left(C_{a}^{b}\right)}^{T}\right]}^{-1}f_{L}$ is the acceleration compensation of dynamic calibration and is composed of direction matrix $C_{a}^{b}$, angle rate $\omega^{b}$ and installation position vector $r^{b}$, namely, dynamic calibration added the $f_{L}^{b}$ compared with static position. We have:

(17) $$f_{L}^{b}={\left[{\left(C_{a}^{b}\right)}^{T}\right]}^{-1}\left[\begin{array}{c} C_{a}^{b}{\left(*,1\right)}^{T}Wr_{X}^{b}\\ C_{a}^{b}{\left(*,2\right)}^{T}Wr_{Y}^{b}\\ C_{a}^{b}{\left(*,3\right)}^{T}Wr_{Z}^{b} \end{array}\right]$$

When ω is a constant value, $\overset{•}{\omega }$ = 0, W is simplified as $W={\left(\omega \times \right)}^{2}$.

### 2.3. Identification of Lever Arm

Installed direction error has been compensated by using the static calibration. Sensing axes of the three accelerometers are perpendicular orthogonal, which are parallel to the carrier frame, respectively. 

When accelerometers rotate at a constant speed of $\omega_{0}$ ($\overset{•}{\omega_{0}}=0$), installation vector is $r_{i}^{b}=(\begin{array}{ccc} r_{ix} & r_{iy} & r_{iz} \end{array})$, for which *i* = 1,2,3 is the number of accelerometer. $r_{x}$, $r_{y}$, $r_{z}$ are vectors along three axes, respectively. As sensing direction of $A_{i}$ is along the input axis, and the error caused by the lever arm is ω×(ω×r) on frame b. In order to identify parameter *r* easily, the following method is used.

If we set $f_{0}^{xb}=\left(\begin{array}{ccc} g & 0 & 0 \end{array}\right)$ and $r_{1}=\left(\begin{array}{ccc} r_{1x} & r_{1y} & r_{1z} \end{array}\right)$, then the output of the three accelerometers are given by:

(18) $$\{\begin{array}{c} f_{x0}^{xa}=C_{a}^{b}{\left(*,1\right)}^{T}f_{0}^{xb},i=x,y,z\\ f_{y0}^{xa}=C_{a}^{b}{\left(*,2\right)}^{T}f_{0}^{xb},i=x,y,z\\ f_{z0}^{xa}=C_{a}^{b}{\left(*,3\right)}^{T}f_{0}^{xb},i=x,y,z \end{array}$$

(19) $$\{\begin{array}{c} f_{x0}^{xa}=\left(\begin{array}{ccc} \cos\theta_{xx} & \cos\theta_{xy} & \cos\theta_{xz} \end{array}\right){\left(\begin{array}{ccc} f_{x0}^{xb} & f_{y0}^{xb} & f_{z0}^{xb} \end{array}\right)}^{T}\\ f_{y0}^{xa}=\left(\begin{array}{ccc} \cos\theta_{yx} & \cos\theta_{yy} & \cos\theta_{yz} \end{array}\right){\left(\begin{array}{ccc} f_{x0}^{xb} & f_{y0}^{xb} & f_{z0}^{xb} \end{array}\right)}^{T}\\ f_{z0}^{xa}=\left(\begin{array}{ccc} \cos\theta_{zx} & \cos\theta_{zy} & \cos\theta_{zz} \end{array}\right){\left(\begin{array}{ccc} f_{x0}^{xb} & f_{y0}^{xb} & f_{z0}^{xb} \end{array}\right)}^{T} \end{array}$$

The equation above is simplified as Equation (20):

(20) $$f_{i0}^{xa}=C_{a}^{b}{\left(*,j\right)}^{T}f_{i0}^{xb}$$

Here, we define *i* = *x*, *y*, *z*, *j* = 1,2, 3, respectively.

Similarly, $f_{i0}^{yb}=\left(\begin{array}{ccc} 0 & g & 0 \end{array}\right)$, $f_{i0}^{zb}=\left(\begin{array}{ccc} 0 & 0 & g \end{array}\right)$, i.e., $f_{i0}^{ya}=C_{a}^{b}{\left(*,j\right)}^{T}f_{0}^{yb},f_{i0}^{za}=C_{a}^{b}{\left(*,j\right)}^{T}f_{0}^{zb}$. Installation vector is $r_{j}=\left(\begin{array}{ccc} r_{jx} & r_{jy} & r_{jz} \end{array}\right),\left(j=1,2,3\right)$.

When the *x*-axis is vertically upward, turntable rotation at a constant angular velocity *ω*_0_ and specific force output in carrier frame is given by:

$$f_{1i}^{a}=C_{a}^{b}{\left(*,j\right)}^{T}f_{1}^{xb}=C_{a}^{b}{\left(*,j\right)}^{T}\left(f_{1}^{xb}+\omega \times \omega \times r_{i}\right)$$

(21) $$\left\{ \begin{array}{l} f_{x}^{xa}=\left[\begin{array}{ccc} \cos\theta_{xx} & \cos\theta_{xy} & \cos\theta_{xz} \end{array}\right]{\left[\begin{array}{ccc} g & -\omega_{0}^{2}r_{1y} & -\omega_{0}^{2}r_{1z} \end{array}\right]}^{T}\\ f_{y}^{xa}=\left[\begin{array}{ccc} \cos\theta_{yx} & \cos\theta_{yy} & \cos\theta_{yz} \end{array}\right]{\left[\begin{array}{ccc} g & -\omega_{0}^{2}r_{2y} & -\omega_{0}^{2}r_{2z} \end{array}\right]}^{T}\\ f_{z}^{xa}=\left[\begin{array}{ccc} \cos\theta_{zx} & \cos\theta_{zy} & \cos\theta_{zz} \end{array}\right]{\left[\begin{array}{ccc} g & -\omega_{0}^{2}r_{3y} & -\omega_{0}^{2}r_{3z} \end{array}\right]}^{T} \end{array}\right.$$

Similarly, when the *y*-axis and *z*-axis are vertically upward on 1 g, the control turntable rotates at a constant angular velocity ω_0_ and specific force outputs in the carrier frame are:

(22) $$\left\{ \begin{array}{l} f_{x}^{ya}=\left[\begin{array}{ccc} \cos\theta_{xx} & \cos\theta_{xy} & \cos\theta_{xz} \end{array}\right]{\left[\begin{array}{ccc} -\omega_{0}^{2}r_{1x} & g & -\omega_{0}^{2}r_{1z} \end{array}\right]}^{T}\\ f_{y}^{ya}=\left[\begin{array}{ccc} \cos\theta_{yx} & \cos\theta_{yy} & \cos\theta_{yz} \end{array}\right]{\left[\begin{array}{ccc} -\omega_{0}^{2}r_{2x} & g & -\omega_{0}^{2}r_{2z} \end{array}\right]}^{T}\\ f_{z}^{ya}=\left[\begin{array}{ccc} \cos\theta_{zx} & \cos\theta_{zy} & \cos\theta_{zz} \end{array}\right]{\left[\begin{array}{ccc} -\omega_{0}^{2}r_{3x} & g & -\omega_{0}^{2}r_{3z} \end{array}\right]}^{T} \end{array}\right.$$

(23) $$\left\{ \begin{array}{l} f_{x}^{za}=\left[\begin{array}{ccc} \cos\theta_{xx} & \cos\theta_{xy} & \cos\theta_{xz} \end{array}\right]{\left[\begin{array}{ccc} -\omega_{0}^{2}r_{1x} & -\omega_{0}^{2}r_{1y} & g \end{array}\right]}^{T}\\ f_{y}^{za}=\left[\begin{array}{ccc} \cos\theta_{yx} & \cos\theta_{yy} & \cos\theta_{yz} \end{array}\right]{\left[\begin{array}{ccc} -\omega_{0}^{2}r_{2x} & -\omega_{0}^{2}r_{2y} & g \end{array}\right]}^{T}\\ f_{z}^{za}=\left[\begin{array}{ccc} \cos\theta_{zx} & \cos\theta_{zy} & \cos\theta_{zz} \end{array}\right]{\left[\begin{array}{ccc} -\omega_{0}^{2}r_{3x} & -\omega_{0}^{2}r_{3y} & g \end{array}\right]}^{T} \end{array}\right.$$

According to Equation (23), installation position vectors can be given as

(24) $$\{\begin{array}{c} r_{jx}=(f_{i}^{xa}-f_{i}^{ya}-f_{i}^{za}-f_{i0}^{xa}+f_{i0}^{za}+f_{i0}^{ya})/2\omega_{0}^{2}\cos\theta_{ix}\\ r_{jy}=(f_{i}^{ya}-f_{i}^{xa}-f_{i}^{za}-f_{i0}^{ya}+f_{i0}^{za}+f_{i0}^{xa})/2\omega_{0}^{2}\cos\theta_{iy}\\ r_{jz}=(f_{i}^{za}-f_{i}^{xa}-f_{i}^{ya}-f_{i0}^{za}+f_{i0}^{ya}+f_{i0}^{xa})/2\omega_{0}^{2}\cos\theta_{iz} \end{array},\;(j=1,\;i=x;\;j=2,\;i=y;\;j=3,\;i=z).$$

We can get values of $r_{1}$, $r_{2}$, $r_{3}$ if $f_{i}^{a},f_{i0}^{a}$ are given by Equation (2). In the procedure of calibration, owing to the direction cosine in the denominator approach zero, so it becomes distortion that results in the amplification of calculation error. We make an angle deviate between the turntable frame and carrier frame, and use the turntable to avoid the problems. Through numerous experiments at high speed, we can obtain mean values and also provide a more accurate calibration result for subsequent compensation.

Actually, $r=\left(\begin{array}{ccc} 0.01 & 0.01 & 0.01 \end{array}\right)$ (unit m), according to $\delta a=\omega^{2}r$, when rotating at a speed of 100°/s, the acceleration error will be 0.03 m/s^2^. If the period of the measurement takes 100 s, the velocity error will be 3 m/s, and displacement error will be 150 m. Namely, the installation position error can not be ignored.

## 3. Experiments

In order to verify the effectiveness of the proposed method, the static and dynamic experiments are carried out. Furthermore, a ground semi-physical experiment is developed, and the experimental results of pre- and pro- compensation are compared. In this system, the zero bias stability, repeatability, and resolution are 1 mg.

### 3.1. Static Calibration Experiment

(1) MIMU, which linked with a real-time acquisition system to collect data, is fixed on the multi-function tri-axial position rate turntable. *x*-axis upward, *y*-axis and *z*-axis point to south and east directions, respectively.
(2) By setting the turntable position, make accelerometer *x* ideal axis at position ±1 g and collect the output data for a minute at each position.
(3) Turntable is set to make the accelerometers *y*-axis and *z*-axis in position ±1 g. Collect the output data for a minute at each position.
(4) According to static parameters calibration model Equation (6), the least squares fitting results can be obtained by using Matlab (version 7.8.0 (R2009a), MathWorks, Natick, MA, USA), which are actual installation angle, zero bias and scale factors of tri-axial accelerometers, respectively.

### 3.2. Dynamic Calibration Experiment

(1) Fix the MIMU on the turntable, set up the turntable to the *x*-axis in the vertical position steady for two minutes.
(2) Power on the acquisition system and collect one-minute stationary data. Then, set the turntable rate to rotate in an invariable angular rate ω_0_ = 100°/s, 150°/s, and 200°/s around the *x*-axis and collect one minute of data after becoming stable.
(3) Set the turntable to the *y*-axis and *z*-axis on the upright position, repeat the first step and record the output of tri-axial accelerometer timely.

### 3.3. Ground Verification Experiment

**Figure 4:**
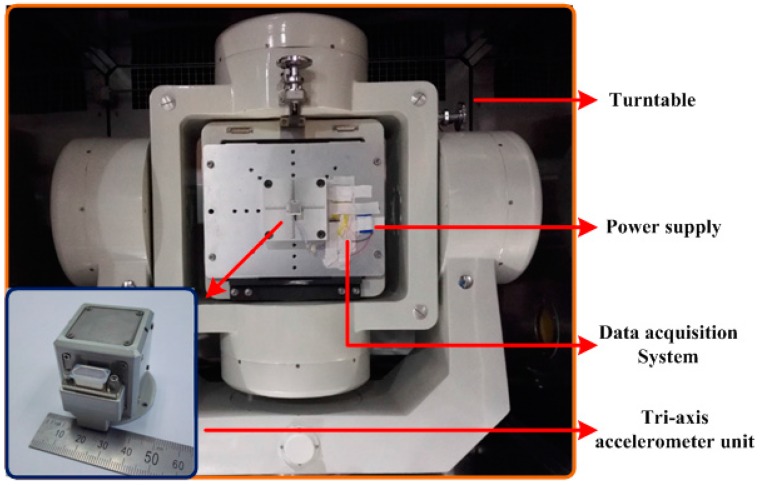
Ground experimental equipment.

To verify the effect of the result of calibration for the navigation measurement accuracy, the ground semi-physical simulation experiment is carried out on a tri-axial rate position turntable as shown in Figure 4. Here, the simulated data is only to show the effectiveness reducing dynamic errors and the simulated data does not include all the error sources. The main equipment includes: (1) high precision three-axis position and velocity turntable; (2) tri-axis accelerometer unit; (3) data acquisition system; and (4) power supply. The type of the accelerometer is MS9005.D (Colibrys, Yverdon-les-Bains, Switzerland), the measurement range is ±5 g, the bias stability is less than 0.75 mg, and the nonlinearity is less than 0.8%.

## 4. Result/Discussion

### 4.1. Static Calibration Result and Discussion

**Figure 5:**
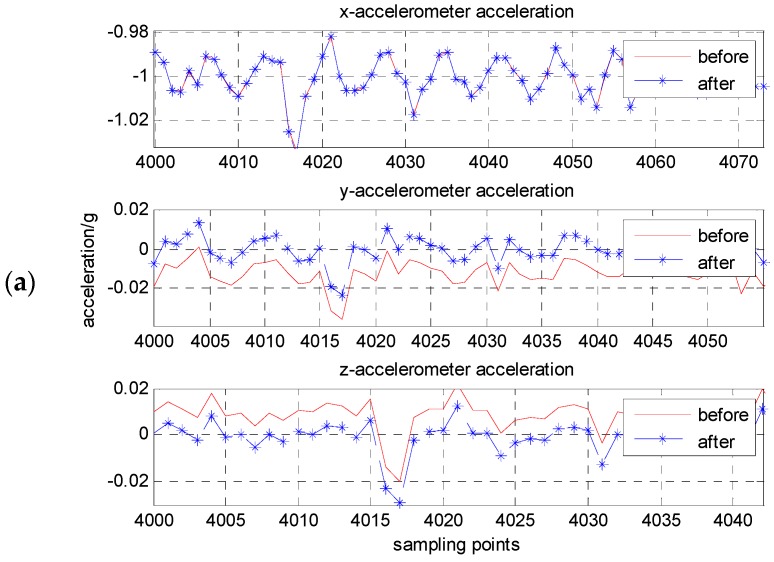

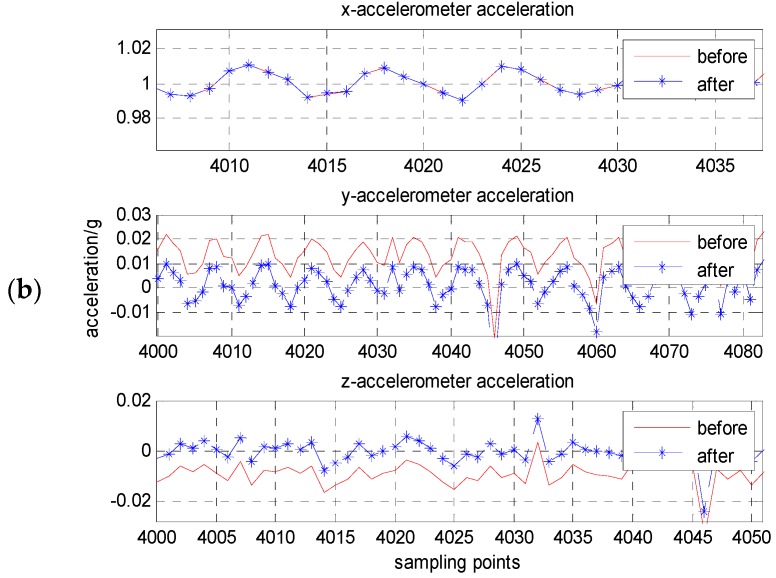
Contrast of three-axis acceleration before and after compensation when the *x*-axis, respectively, at ±1 g position: (**a**) *x*-axis at −1 g position; (**b**) *x*-axis at +1 g position.

Static and calibration results of accelerometer unit are given in Table 1. Figure 5 is a contrast of the accelerometer outputs’ pre- and pro- calibration and compensation by using the proposed method. Table 2 gives the outputs of three-axis acceleration pre- and pro- compensation when the *x*-axis is at the ±1 g position. The absolute error of the accelerometer outputs is less than 1 mg after compensation, and the measurement accuracy is increased by one or two magnitude.

**Figure 6:**
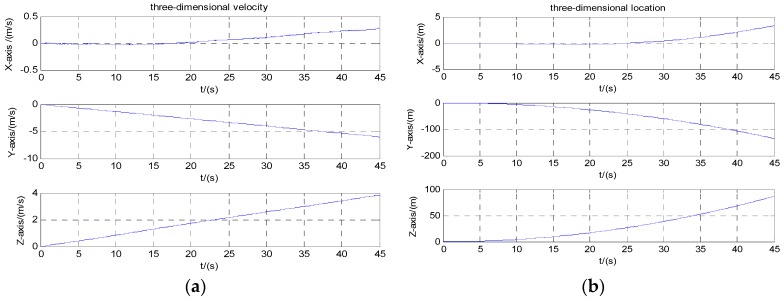
Velocity and position before compensation: (**a**) velocity; (**b**) position.

**Figure 7:**
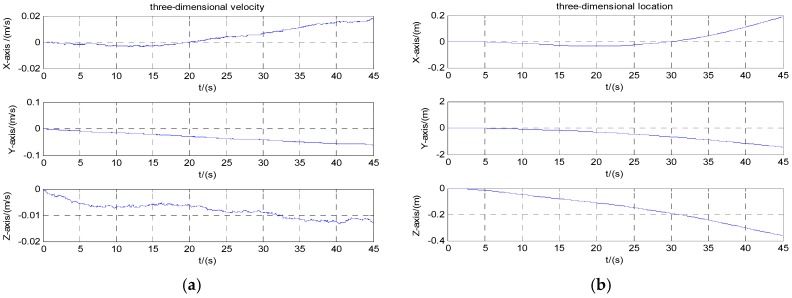
Velocity and position after compensation: (**a**) velocity; (**b**) position.

Navigation calculation starts with initial speed zero. Figure 6a,b are three-dimensional velocity and position information of the carrier before compensation. Figure 7a,b are three-dimensional velocity and position information of the carrier after the calibration compensation. It can be seen from the figures that the velocity error reduced from 4 to 0.02 m/s after calibration and compensation, and the position error reduced from 100 to 0.3 m.

### 4.2. Dynamic Calibration Result and Discussion

**Figure 8:**
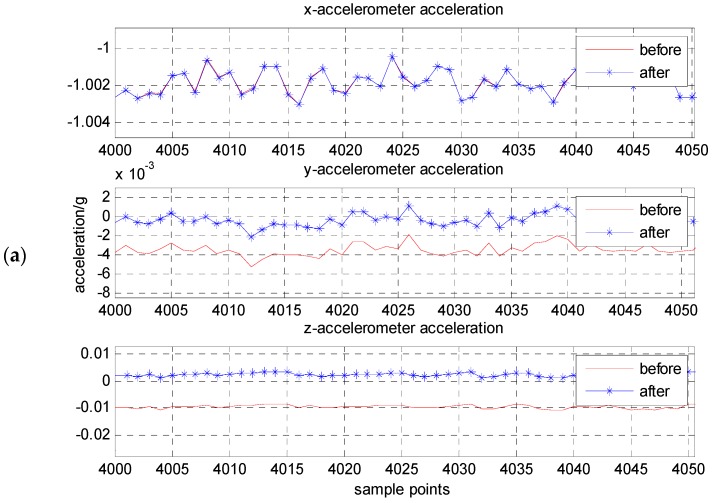

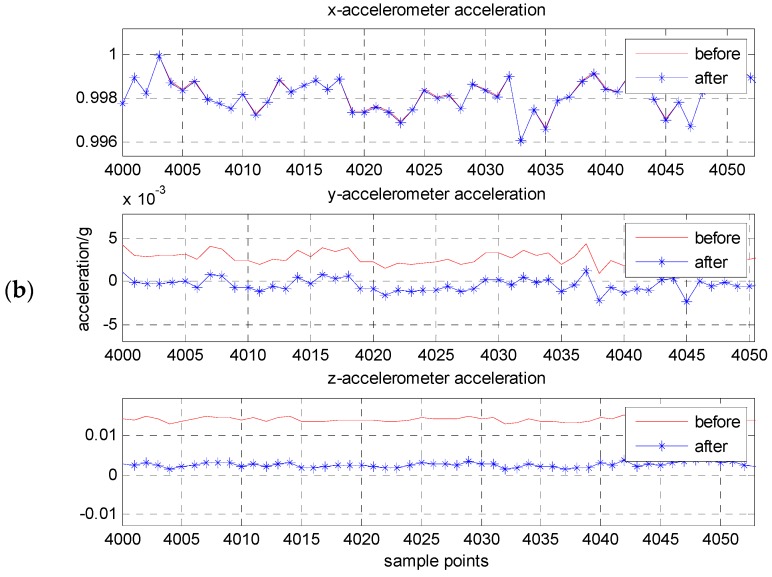
The contrast of three-axis acceleration before and after compensation when the *x*-axis, respectively, at ±1 g position: (**a**) *x*-axis at −1 g position; (**b**) *x*-axis at +1 g position.

Similarity to the results of static calibration described above, Figure 8 is a contrast of the accelerometer outputs’ pre- and pro- calibration and compensation by using the proposed method. The result of dynamic calibration for the accelerometer unit is given in Table 3. Table 4 gives the outputs of three-axis acceleration pre- and pro- compensation when the *x*-axis at ±1 g position. The absolute error of the accelerometer outputs is less than 1 mg after compensation, and the measurement accuracy is increased by one or two magnitude. 

**Figure 9:**
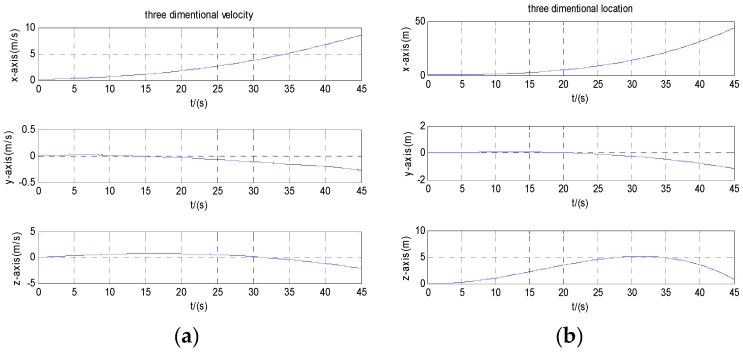
Velocity and position before compensation: (**a**) velocity; (**b**) position.

**Figure 10:**
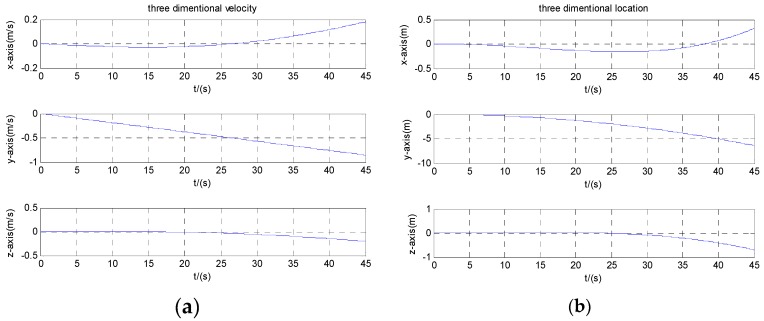
Velocity and position after compensation: (**a**) velocity; (**b**) position.

To illustrate the performance of the proposed dynamic calibration method, Figure 9 and Figure 10 depict the three-dimensional velocity and position information of the carrier before and after compensation. Because of the dynamic calibration and compensation of accelerometer, it can be seen that the system’s velocity and position error is significantly reduced.

## 5. Conclusions

We developed a static and dynamic compensation method for an accelerometer unit, which can make the measuring accelerometer equivalent to a point. In this calibration method, 15 parameters for a tri-axis accelerometer unit were used. Besides the zero bias voltage, the other parameters have different physical significance in contrast with the existing calibration methods. 

The proposed calibration method can effectively remove the dimension effect in dynamic environment. The turntable and semi-physical simulation experiments have to be performed to verify the validity of this dynamic method. The results show that the navigation precision of tri-axis accelerometers can be largely improved at high speed after compensation.

The future work is to reduce the inertial navigation error accumulated along with time for IMU.

## Figures and Tables

**Table 1:** Static calibration of accelerometer unit.

No.	Zero Bias (V)	Scale Factor (V/g)	Installation Angle (Degree)
1	2.405098	0.39496	(0.75997, 90.71425, 89.74038)
2	2.380978	0.39808	(89.25516, 0.74486, 90.5630)
3	2.435503	0.40024	(90.52202, 89.17706, 0.97456)

**Table 2:** Output of tri-accelerometer unit when the accelerometer of the *x*-axis is ±1 g (unit: g).

Sensor	Theoretical Output	Before Compensation	After Compensation	Before Compensation	After Compensation
*x*	±1 g	1.00043	1.00041	−0.99956	−0.99958
*y*	0 g	0.01299	0.00098	−0.01100	0.00098
*z*	0 g	−0.00911	0.00023	0.00953	0.00020

**Table 3:** Dynamic calibration for accelerometer unit.

Installation Position Vector		Installation Position Vector (m)	
r1	−0.00834	−0.00042	−0.00132
r2	−0.00256	−0.00893	−0.00026
r3	−0.00060	−0.00050	−0.00824

**Table 4:** Output of the tri-accelerometer unit when the accelerometer of the *x*-axis is ±1 g. (unit: g).

Sensor	Theoretical Output	Before Compensation	After Compensation	Before Compensation	After Compensation
*x*	±1 g	0.998078	0.998060	−1.001924	−1.001939
*y*	0 g	0.000289	0.000253	−0.003359	0.000253
*z*	0 g	−0.014150	0.002402	0.0093985	0.002402
